# Essential Oils of *Mentha arvensis* and *Cinnamomum cassia* Exhibit Distinct Antibacterial Activity at Different Temperatures In Vitro and on Chicken Skin

**DOI:** 10.3390/foods12213938

**Published:** 2023-10-27

**Authors:** Iglė Vepštaitė-Monstavičė, Bazilė Ravoitytė, Jurga Būdienė, Algirdas Valys, Juliana Lukša, Elena Servienė

**Affiliations:** 1 Laboratory of Genetics, Nature Research Centre, 08412 Vilnius, Lithuania; igle.vepstaite-monstavice@gamtc.lt (I.V.-M.); algirdas.valys@gamtc.lt (A.V.); juliana.luksa@gamtc.lt (J.L.); 2 Laboratory of Chemical and Behavioural Ecology, Nature Research Centre, 08412 Vilnius, Lithuania; jurga.budiene@gamtc.lt; 3 Department of Chemistry and Bioengineering, Vilnius Gediminas Technical University, 10223 Vilnius, Lithuania

**Keywords:** *Cinnamomum cassia*, *Mentha arvensis*, essential oils, *Salmonella typhimurium*, essential oil vapor, antibacterial activity, temperature

## Abstract

The bacterial contamination of meat is a global concern, especially for the risk of *Salmonella* infection that can lead to health issues. Artificial antibacterial compounds used to preserve fresh meat can have negative health effects. We investigated the potential of natural essential oils (EOs), namely *Mentha arvensis* (mint) and *Cinnamomum cassia* (cinnamon) EOs, to prevent contamination of the food pathogen, *Salmonella enterica* subsp. *enterica* serotype Typhimurium, in vitro and on chicken skin. The gas chromatography–mass spectrometry (GC-MS) technique was used to determine the compositions of mint EO (MEO) and cinnamon EO (CEO); the most abundant compound in MEO was menthol (68.61%), and the most abundant compound was cinnamaldehyde (83.32%) in CEO. The antibacterial activity of MEO and CEO were examined in vapor and direct contact with *S. typhimurium* at temperatures of 4 °C, 25 °C, and 37 °C. The minimal inhibitory concentration at 37 °C for MEO and CEO reached 20.83 µL/mL, and the minimal bactericidal concentration of CEO was the same, while for MEO, it was two-fold higher. We report that in most tested conditions in experiments performed in vitro and on chicken skin, CEO exhibits a stronger antibacterial effect than MEO. In the vapor phase, MEO was more effective against *S. typhimurium* than CEO at 4 °C. In direct contact, the growth of *S. typhimurium* was inhibited more efficiently by MEO than CEO at small concentrations and a longer exposure time at 37 °C. The exploration of CEO and MEO employment for the inhibition of *Salmonella* bacteria at different temperatures and conditions expands the possibilities of developing more environment- and consumer-friendly antibacterial protection for raw meat.

## 1. Introduction

EOs are hydrophobic extracts, which are rich in plant secondary metabolites, and they are intensively studied for their antimicrobial and other valuable properties. EOs extracted from mint, cinnamon, clove, basil, lavender, thyme, sage, etc., have GRAS (Generally Recognized as Safe) statuses provided by the Food and Drug Administration (FDA) and are considered to be safe for human consumption. Researchers have successfully demonstrated the potential of several plant EOs for the microbiological protection of various food products [1,2,3]. The antibacterial activity of an EO is mostly determined by the main components of the EO; however, an EO usually has stronger antibacterial properties than its main components alone [4]. EOs from plants, including mint and cinnamon, have been shown to inhibit *Salmonella*, which is one of the most common foodborne pathogens [5,6]. Synthetic additives and food preservatives are used to eliminate or suppress the growth of bacteria in food products; however, the consumption of these substances may have negative health effects for consumers [7]. Thus, there is a high demand for natural additives with efficient antibacterial properties in the food industry, especially regarding ecological food and environmental awareness [8,9,10,11].

The properties of EOs, mainly their high hydrophobicity and volatility, make the investigation challenging. The efficacy of EOs and their individual constituents on the survival of microorganisms has been studied extensively by measuring the effects of direct contact with the oils and the impact of EO vapor. However, there is still a lack of standard testing procedures, especially for the investigation of the vapor phase, but attempts to standardize the methods are emerging [12,13]. Temperature is an insufficiently investigated ambient element that affects the action mechanism of an EO [14,15,16]. Temperature has impacts on the cellular processes and the viscosity of lipid membranes and the release and evaporation properties of EO compounds, thereby conditioning the interaction between EO components and the cellular membrane [14,15,16]. The main mechanism of EO action is the destabilization and permeabilization of the bacterial cell membrane [16]. Recent research has elucidated the effects of cinnamaldehyde, menthone, and CEO against *Salmonella* and *Staphylococcus* bacteria by analyzing transcriptional responses, cell membrane properties, oxidative damage, and metabolism [17,18,19,20,21]. However, precise knowledge of the action mechanisms remains relatively scant, especially regarding the antimicrobial potentials of EOs in food products [22,23].

MEO has a wide range of positive health effects and is used for the treatment of various skin infections, headaches, and respiratory and gastrointestinal tract problems [24,25]. The main components of MEOs are menthol, neomenthol, menthone, isomenthone, carvone, limonene, linalool, geraniol, pulegone, menthyl acetate, and others [25]. Menthone has antibacterial properties and was shown to inhibit the growth of methicillin-resistant *Staphylococcus aureus* [18]. EOs, extracted from leaves of *Mentha* genus plants, and their individual components have shown inhibitory activity towards various bacteria [22,26,27], yeasts [28], fungi [29], and insects [30].

CEO has strong broad-spectrum antibacterial, antifungal, antioxidant, and insecticidal activities [4,31]. Cinnamaldehyde is the main constituent of CEO extracted from the tree bark regardless of the cinnamon species and accounts for approximately 65–80% of CEO [32]. It inhibits the growth of many tested bacteria, including *S. aureus* and *S. typhimurium* [32,33], and has been recently shown to suppress the ability of *S. typhimurium* to adhere to cells, thus reducing the possibility of infection [20]. Other common CEO components are eugenol, benzaldehyde, cinnamyl acetate, *α*-pinene, and linalool [31,34]. The mechanisms of the effects of CEOs seem to be complex since the constituents of the oil have different functional groups and may act via different mechanisms to disturb the functioning of the target cells [32].

The aim of this work is to investigate the antibacterial activity of CEO and MEO via vapor and direct contact with *S. typhimurium* bacteria in vitro and on chicken skin at different temperatures, namely 4 °C, 25 °C, and 37 °C. The main compositions of MEO and CEO were determined via GC-MS. The minimal inhibitory concentrations (MICs) and minimal bactericidal concentrations (MBCs) of MEO and CEO in direct contact with *S. typhimurium* were established. The EO vapor’s antibacterial activity against *S. typhimurium* and *Staphylococcus aureus* bacteria was tested at different temperatures in vitro, and CEO showed greater inhibition than MEO. In the vapor phase, the efficacy of CEO against *S. typhimurium* on chicken skin was greater than the activity of MEO.

## 2. Materials and Methods

### 2.1. Essential Oils

EOs of wild mint (*Mentha arvensis*) and Chinese cinnamon bark (*Cinnamomum cassia*) used in this research were bought from one retailer in Lithuania, JSC Kvapu namai. Both EOs were labeled with traditional and botanical names of the plant they were produced from via hydrodistillation, the series number, country of origin, and expiry date (Table 1).

### 2.2. EO Chemical Composition Analysis Using GC-MS

GC-MS was applied to determine the chemical compositions of MEO and CEO. A total of 10 µL of each EO was dissolved in 1 mL of pentane (≥99% (GC), Sigma-Aldrich, Darmstadt, Germany) and diethyl ether (containing 1 ppm BHT as inhibitor, anhydrous, ≥99.7% (GC), Sigma-Aldrich, Darmstadt, Germany) (1:1) mixture. An amount of 1 µL of the prepared solution was injected into the GC/MS system. Analyses were performed using Shimadzu GC/MS-Q2010 PLUS interfaced to a GC-MS-QP2010 ULTRA mass spectrometer (Shimadzu, Kyoto, Japan) and fitted with a non-polar Rxi-5MS (30 m × 0.25 mm × 0.25 µm) (Restek, Bellefonte, PA, USA) capillary column. Mass spectra in electron impact mode were generated at 70 eV, with 0.97 scans per second and a mass range of 33–400 *m*/*z*. The oven temperature of the gas chromatograph was set at 50 °C (for 1 min) and then increased by 5 °C per minute to reach 160 °C; then, it was held for 2 min and programmed to reach 250 °C at the increasing rate of 10 °C/min and was held at the final temperature for 4 min. He with a flow rate of 1.0 mL/min was used as carrier gas. Detector and injector temperatures were 250 °C, and the ion source temperature was 220 °C. The qualitative analysis and identification of compounds were based on the comparison of time and retention indexes of the column, with corresponding data in the literature [35], as well as computer libraries of mass spectra (using “GC/MS solution” v. 2.71 software from Shimadzu and Wiley and NIST). The identification of the compound was approved if the mass spectra library data matched the computer data with a probability equal to 90% or above. The retention indices were determined regarding retention times of a series of n-alkanes (C_7_–C_30_) (≥99% (GC), Supelco, Bellefonte, PA, USA) with linear interpolation. The relative percentage of EO constituents was computed from the chromatogram peak areas with none of the correction factors.

### 2.3. Bacterial Cultures and Media

Bacterial strains *Salmonella enterica* subsp. *enterica* serotype Typhimurium LT2 (kindly provided by Jaunius Urbonavičius, Vilnius Gediminas Technical University, Lithuania) and *Staphylococcus aureus* ATCC 29213 (kindly provided by Eglė Lastauskienė, Vilnius University, Lithuania) were used in this study. Bacteria were grown on LB agar (1% peptone (Liofilchem, Roseto degli Abruzzi, Italy), 0.5% yeast extract (Liofilchem, Roseto degli Abruzzi, Italy), 0.5% NaCl (≥99.5%, Carl Roth, Karlsruhe, Germany), 2% agar (Liofilchem, Roseto degli Abruzzi, Italy) on solid medium plates at 37 °C. Freshly grown cells were inoculated in LB broth and grown overnight (16–18 h) at 37 °C with continuous shaking. The optical density (OD) of cells was determined spectrophotometrically (600 nm), and the cell number was adjusted to an OD of 1 in LB broth medium. Reagents from Liofilchem, Italy were microbiological grade.

### 2.4. Antibacterial Activity Testing in Agar Plates

To determine the antibacterial activity of EO in agar plates, 20 µL of *S. typhimurium* cells (about 1 × 10^7^ cells/plate) was evenly spread onto the plates with LB agar medium. For the measurement of direct contact between the cells and EO, 10 µL of pure EO was placed on the surface of the LB agar medium covered with bacteria. The antibacterial effect of EO vapor was tested using the inverted Petri dish method. A total of 25 µL of pure EO was placed on the inside surface of the plate cover, and LB agar plates with bacteria were covered and sealed with parafilm. Plates were incubated at 4 °C, 25 °C, and 37 °C overnight (24 h). Three plate replicates of each condition were tested. After incubation, the size of the growth inhibition zone was evaluated.

### 2.5. Determination of MIC and MBC

To determine MIC, 120 µL of *S. typhimurium* cells (about 1 × 10^7^ cells/mL) was mixed with an equal amount of the suspension containing EO to achieve the final concentration of 5 × 10^5^ cells/well. Suspension containing EO consisted of EO premixed with 75% ethanol (food grade, 96%, Vilniaus degtinė, Vilnius, Lithuania) at a 1:1 ratio (*v*/*v*), diluted with LB broth. Control samples containing concentrations of ethanol corresponding to EO samples were used to confirm that the amount of alcohol added does not have a significant impact on the survival of cells. Tested final concentrations of EO varied from 0.008% to 12.5% and for ethanol—from 0.006 to 9.37% per well. Samples were incubated at 37 °C overnight (24 h). The following day, the wells with bacterial suspensions were visually inspected, and the smallest concentration of EO at which no signs of bacterial growth were observed (liquid turbidity or cell sedimentation) was considered the MIC. To determine MBC, 25 µL of bacterial suspension in the wells was distributed on plates with LB agar medium and incubated at 37 °C overnight (24 h). The following day, the growth of bacteria in each plate was evaluated. The smallest concentration of EO that reduced the number of viable *S. typhimurium* cells by more than 99% (i.e., less than 125 colonies per plate) was considered the MBC.

### 2.6. S. typhimurium Killing upon Direct Contact with EO In Vitro

To investigate the rate of the inhibition of bacterial survival via direct contact with the EO, several EO concentrations at different temperatures were tested. Each EO was premixed with 75% ethanol at a 1:1 ratio (*v*/*v*). The MIC of each EO was diluted 10-fold, 100-fold, and 1000-fold with LB broth. In control samples, EO was replaced with an equal amount of sterile distilled water. Each EO dilution at equal volume was mixed with 100 µL of *S. typhimurium* cells (final concentration 1 × 10^7^ cells/mL) and incubated for 10, 20, and 40 min at temperatures of 4 °C, 25 °C, and 37 °C. Serial dilutions were made, and 50 µL of each solution was spread onto the LB agar plates and incubated at a 37 °C temperature for 24 h. Colonies on plates were counted as colony-forming units (CFUs), and the mean values of CFU/mL were calculated.

### 2.7. Antibacterial Activity of EO Vapor In Vitro

EO vapor antibacterial activity was tested against Gram-negative *S. typhimurium* and Gram-positive *S. aureus* bacteria. To determine the antibacterial activity of EO vapor in different temperatures, 120 µL of cells at OD 1 was mixed with an equal amount of LB broth in 1.5 mL volume microcentrifuge tubes (Eppendorf Safe-Lock), and 25 µL of pure EO was placed into the inner side of the tube cap. In control samples, LB broth was used instead of EO. Tubes were positioned horizontally to avoid contact between the cells and the EO. Tubes were incubated at temperatures of 4 °C, 25 °C, and 37 °C for 1 h, 4 h, 24 h, 48 h, and 120 h. Serial dilutions were made, and 50 µL of each solution was spread onto the LB agar plates and incubated at a 37 °C temperature for 24 h. All experiments were performed in triplicate. The number of survived cells in each sample was calculated, and the results were expressed as mean value lg (CFU/mL) ± standard deviation.

### 2.8. Antibacterial Activity of EO Vapor on Chicken Skin

Fresh chicken pieces were bought from the local supermarket in Vilnius, Lithuania. The chicken skin was removed from the flesh and cut into 2 cm × 2 cm pieces. The skin was rinsed with cold running tap water and with sterile distilled water. The excess water from the washed skin was removed via absorption with sterile paper. Dry pieces of chicken skin were placed onto sterile Petri dishes and sterilized with a UV dose of 12 mJ/cm^2^. On each skin sample, 25 µL of *S. typhimurium* (1 × 10^7^ cells/mL) was placed. Plates were incubated at 4 °C for 30 min to enhance the adhesion of bacteria on the skin’s surface. In the center of the inner Petri dish cover, 25 µL of the pure EO was placed. In the control plates, a sterile 0.9% NaCl solution was used instead of EO. Plates were covered with lids and insulated with parafilm. Plates were incubated at temperatures of 4 °C, 25 °C, and 37 °C for 1 h, 4 h, 24 h, 48 h, and 120 h. After incubation, skin samples were transferred to sterile 50 mL conical centrifuge tubes. To each tube, 15 mL of sterile 0.9% NaCl solution was added, and tubes were gently inverted continuously for 5 min to detach the bacteria from the skin’s surface. Serial dilutions were made, and 50 µL of each solution was spread onto the LB agar plates, followed by overnight incubation at 37 °C and counting of colony-forming units (CFUs). Experiments were performed in triplicate, and the mean value of CFU/mL was calculated.

### 2.9. Statistical Analysis

A one-way analysis of variance (ANOVA, *p* < 0.05) was used to define statistically significant results. All experiments were performed in triplicate, and the treatment efficiency is expressed as mean ± standard deviation. Statistical analysis was carried out using Microsoft Office Excel.

## 3. Results and Discussion

### 3.1. Chemical Compositions of C. cassia and M. arvensis EOs

MEO and CEO are safe, economically important, and relevant for use in the food industry, including applications in food packaging [36], and thus were selected for the investigation. The gas chromatography–mass spectrometry technique was used for the qualitative chemical analysis of MEO and CEO. The main compounds of the EOs are presented in Table 2, and their full composition is available in the Supplementary Materials (Table S1).

Both EOs used in the study contained one predominant compound (menthol in MEO and (*E*)-cinnamaldehyde in CEO), accounting for more than 50% of the total compounds identified (Table 2). In MEO, the second and third most abundant compounds were menthone (7.9%) and isomenthone (6.42%), and in CEO, the second and third most abundant compounds were (*E*)-methoxy-cinnamaldehyde (7.62%) and (*E*)-cinnamyl acetate (4.69%), respectively. In the latter, all other identified compounds were present in very small quantities, not exceeding 1%. In MEO, *α*-pinene, *β*-pinene, limonene, geraniol, and menthyl acetate were present in amounts exceeding 1% of the total composition, with other compounds being present in minor or trace levels. When comparing the compositions of the two EOs, it should be noted that there are only a few compounds in common between them, and these compounds are present in very modest amounts. Both predominant compounds, in addition to being plant-specific odorants, have strong antifungal, insecticidal, and antibacterial properties [4,37,38].

The main constituents of MEO and CEO are terpenes and terpenoids. The most dominant groups in MEO are monoterpenoid menthol and monoterpenes, while in CEO, phenylpropanoids prevailed. Our findings are consistent with previous research, since menthol is the main component in MEO, and cinnamaldehyde is the main component in CEO [25,31,34,39]. The antioxidant and antibacterial properties of whole EOs are usually stronger than those of individual components since constituents in trace amounts act in synergy [4]. The compositions of MEO and CEO are promising in terms of antibacterial potential.

### 3.2. CEO and MEO Antibacterial Activity against S. typhimurium in Agar Plates

Gram-negative *S. typhimurium* was selected for the evaluation of the antibacterial activity of the EOs since it is one of the most prevalent food pathogens worldwide [40]. It has already been demonstrated that various EOs can inhibit the proliferation of *Salmonella* bacteria in vitro and in various food products [6,15,41]. We aimed to explore the potentials of MEO and CEO vapor and oils in inhibiting the growth of *S. typhimurium* at different temperatures and distinct experimental environments.

**Figure 1:**
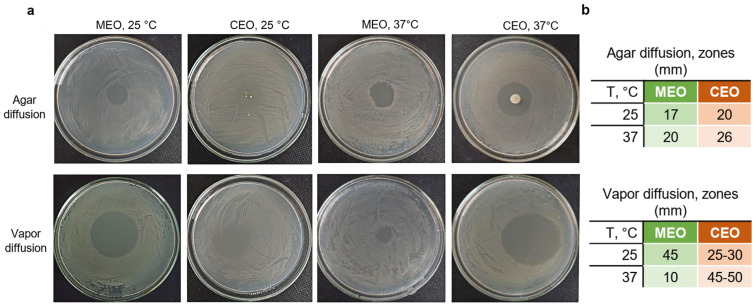
*S. typhimurium* growth inhibition via agar diffusion and vapor diffusion of MEO and CEO. (**a**) *S. typhimurium* growth inhibition at 25 °C and 37 °C. (**b**) Diameters of inhibition zones at 25 °C and 37 °C.

Experiments on agar plates confirmed that CEO exhibits stronger antibacterial activity against *S. typhimurium* than MEO (Figure 1a,b). This effect was profound in both types of experiments, namely agar diffusion (when cells in agar medium were in direct contact with EOs) and vapor diffusion (when cells were in contact with EO vapor). The only exception when MEO exhibited a stronger inhibition of *S. typhimurium* growth than CEO was in the vapor phase at 25 °C, where diameters of clear zones were 45 mm and 25–30 mm, respectively (Figure 1b). *S. typhimurium* growth at 4 °C was not sufficient to obtain visible results; thus, we further describe only the results of the experiments performed at 25 °C and 37 °C.

The growth inhibition zones were generally greater in the vapor diffusion experiments for both EOs in comparison to agar diffusion; however, MEO vapor was less effective at 37 °C, showing half of the diameter of the inhibition zone of the direct contact (10 mm and 20 mm, respectively). In the agar diffusion experiments of MEO and CEO, and in the vapor diffusion experiment for CEO, the antibacterial effect was stronger at 37 °C than at 25 °C (Figure 1b). The MEO vapor at 37 °C demonstrated the lowest *S. typhimurium* growth inhibition from all the tested conditions. The disk diffusion method conducted at 28 °C with 1 µg of EO per disk showed 17–18 mm inhibition zones of *S. typhimurium* induced by MEOs from *Mentha piperita* and *Mentha spicata*, while individual components, namely menthol, limonene, *α*-pinene, and *β*-pinene, created inhibition zones of 18, 8, 10, and 8 mm, respectively [42]. CEO and cinnamaldehyde have shown inhibition zones of 16.3 mm and 14.4 mm for *S. enteritidis*, and 27.7 mm and 23.5 mm for *Salmonella gallinarum* at 37 °C [43], respectively.

The agar diffusion experiments previously demonstrated the antibacterial effect of MEO against *Salmonella* and other bacteria [14,44]. A previous study evaluated the effects of several individual compounds of MEO at 37 °C that are also present in the MEO in our study, namely menthol, menthone, menthyl acetate, limonene, and *β*-pinene [44]. Based on previous data, menthol is the strongest growth inhibitor, whereas *β*-pinene was shown to have little to no impact on *Salmonella* sp. propagation [42,44]. Thus, menthol must not only be the most abundant compound in MEO but also the major determinant of the antibacterial properties of MEO. An *M. piperita* EO addition to model food systems incubated at 4 °C and 10 °C showed diminished *S. enteritidis* numbers during a period of one week [14], confirming the antibacterial properties of mint EO at lower temperatures. CEO has also shown antibacterial effects against *Salmonella* spp. in several foods, such as tahini [15]. CEO is often described as one of the most effective EOs in many studies investigating antibacterial properties [45]. According to one study [15], in a disk diffusion assay, CEO showed the highest growth inhibition of *Salmonella* spp. at 37 °C and 10 °C, where it was also tested, and it was lower at 25 °C, which is consistent with our findings. CEO has a prominent antibacterial effect, which is determined by CEO components, mainly cinnamaldehyde, since the aldehyde functional group provides strong antibacterial properties, while cinnamyl acetate shows lower activity than other major CEO components [46]. *Methoxy*-cinnamaldehyde likely has similar antibacterial properties to cinnamaldehyde because of the aldehyde group, although to our knowledge, this has not been demonstrated yet; however, *o-methoxy*-cinnamaldehyde has been proven to have antifungal activity against pathogenic *Candida* species [47]. The MIC values for *Salmonella* sp. and *Candida albicans* were the same for several MEO components, supporting the efficacy of the tested oils not only against bacteria but also against yeasts [44].

The optimal growth of *S. typhimurium* is observed at 35–37 °C, while typically, it can still grow at lower rates in the range between 5 and 47 °C on chicken meat [48]. Temperature affects the growth rate of bacteria and the properties of the cell membrane, thereby altering the state of the cell and the interaction with EO components [16]. Temperature also has an impact on the antibacterial activity of EO vapor by regulating the vapor pressure and the release of antibacterial volatiles [49]. We demonstrated that both MEO and CEO vapor and oil show an effective inhibition of *S. typhimurium* growth on agar plates at 25 °C and 37 °C, likely as a result of both the action of EO and the speed of growth of the cells at particular temperatures.

### 3.3. S. typhimurium Cells Killed during Direct Contact with MEO and CEO In Vitro

The MIC and MBC for *S. typhimurium* were determined at 37 °C. The MICs of MEO and CEO were 20.83 µL/mL, while the MBC for CEO was the same as the MIC, and for MEO, it was 41.67 µL/mL, two-fold higher. Because of differences in the compositions of EOs, the comparison between their MIC values is complicated; thus, the indicative range of the MICs of EOs and their components is relevant. The reported MIC at 28 °C of the main component in MEO—menthol—for *S. typhimurium* was 1.0 µg/mL, and the MBC was 1.5 µg/mL, while for limonene, *α*-pinene, and *β*-pinene, the MIC and MBC values were between 8.0 and 10.0 µg/mL [42]. The established MIC value for *Salmonella* sp. at 37 °C of menthol was 6 µg/mL, while for menthone, menthyl acetate, and limonene, the value was 10-fold greater—60 µg/mL [44]. CEO was reported to show an MIC value of 0.02% for *S. enteritidis* and *S. gallinarum* at 37 °C [43]. The MIC values of CEO and cinnamaldehyde for *Salmonella* spp., as published by most authors, is about 0.32–1 mg/mL [17].

**Figure 2:**
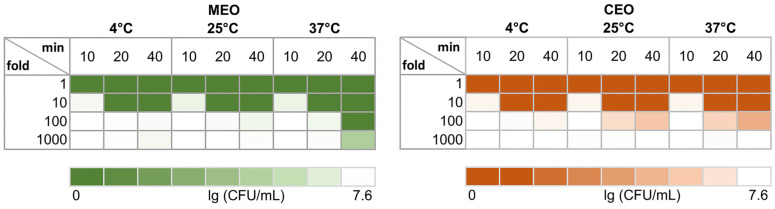
Viability of *S. typhimurium* bacteria after incubation with MEO and CEO at different temperatures. Each EO was premixed with 75% ethanol at a 1:1 ratio (*v*/*v*). MIC of each EO was diluted by 10-fold, 100-fold, and 1000-fold with LB broth.

To better understand the dynamics of *S. typhimurium* growth inhibition by EOs at different temperatures, the MICs at 37 °C were selected as reference points for the concentrations to be tested. Serial dilutions of EOs at a 20.83 µL/mL concentration were tested at 4 °C, 25 °C, and 37 °C at different timepoints, namely 10, 20, and 40 min. As fast as after 10 min, at all tested temperatures, both EOs at 20.83 µL/mL (1-fold dilution) killed all the cells—7.6 logs (Figure 2; Table S2).

No viable cells were detected for both EOs starting at 10-fold dilutions (2.08 µL/mL) as soon as after 20 min of exposure, regardless of the temperature. After 10 min, MEO inhibited the growth of about 3.2-fold more cells than CEO at 25 °C and 37 °C. CEO at a 100-fold dilution (0.21 µL/mL) at 25 °C exhibited 100 and 1000 times higher antibacterial activity after 20 and 40 min, respectfully, than MEO. At 37 °C, CEO’s antibacterial activity after 20 and 40 min incubation was only up to 7-fold higher than at the corresponding timepoints at 25 °C, whereas MEO showed a robust increase in killing, and there were no viable cells after 40 min, which was even 3 logs less than the amount of cells seen in the CEO samples at the same EO concentration. MEO also exhibited significant killing at the lowest concentration tested—1000-fold dilution (0.021 µL/mL)—where only 3 logs out of 7.6 logs of live cells remained at 37 °C. Previous research has demonstrated that EOs from distinct plants, despite having the same MIC values, can have different inhibition mechanisms, as it was in the case of *S. typhimurium* treated with sublethal concentrations of cinnamon and thyme EOs [17]. It may be the case for the CEO and MEO as well, since these oils have different compositions.

At 4 °C and at the highest concentrations (MIC and 10-fold dilution) at all tested temperatures, both EOs have similar antibacterial activity against *S. typhimurium*. At 25 °C, CEO shows more efficient killing than MEO at a lower concentration (100-fold dilution). However, at 37 °C after 40 min of exposure, MEO shows significantly stronger killing than CEO at lower concentrations (100-fold and 1000-fold dilution), where the antibacterial activity with a 10 times smaller concentration than that of MEO equals that of more concentrated CEO (Figure 2; Table S2). A similar approach was used in previous work to test *S. typhimurium* growth kinetics during the exposure to thyme and cinnamon EOs [17], which showed that both EOs were able to prolong the lag phase, both at different intensities, despite the same MIC values for both EOs. Our results also show a similar difference, and it may result from the distinct mechanisms of the EOs’ actions, and the ability and time required for cells to cope with the EO-induced damage [17,50]. CEO and MEO are almost equally effective at 4 °C and for up to 20 min of contact with cells at 25 °C. This may be related not only to the composition and mechanism of action of an EO but also to the slower growth rate and different susceptibility of bacteria at lower temperatures. The greatest effectivity difference is observed after 40 min of incubation, especially at 37 °C; at 25 °C, CEO is more effective than MEO, and vice versa at 37 °C.

### 3.4. Effects of MEO and CEO Vapors on the Viability of S. typhimurium and S. aureus

**Figure 3:**
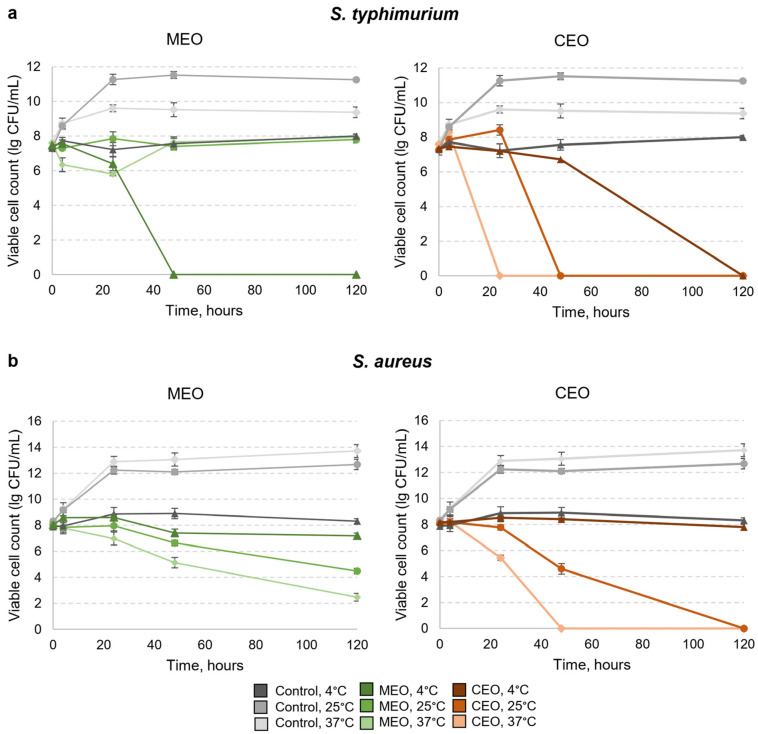
Effects of vapors of MEO and CEO on growth of *S. typhimurium* and *S. aureus* bacteria at 4 °C, 25 °C, and 37 °C. Viable *S. typhimurium* (**a**) and *S. aureus* (**b**) cell counts after exposure to MEO and CEO vapors. All statistical analysis data are provided in Table S3.

We initially aimed to investigate the effects of MEO and CEO vapors on the viability of *S. typhimurium* cells in vitro. The viable cell count was measured at 4 °C, 25 °C, and 37 °C at different timepoints for 120 h. The growth of *S. typhimurium* cells exposed to MEO vapor at 25 °C and 37 °C was similar to the control cell growth at 4 °C with no EO (Figure 3a), where the cell count remained generally stable. The CEO vapor’s activity was more diverse based on the incubation temperature where no viable *S. typhimurium* cells were detected after 24 h at 37° C, 48 h at 25 °C, and 120 h at 4 °C (Figure 3a).

The strongest *S. typhimurium* growth inhibition by the MEO vapor was observed after 48 h of exposure at 4 °C, where no cells were viable. This result was unexpected; thus, we decided to test whether MEO vapor has such a strong inhibitory activity only against *Salmonella* as Gram-negative or if it may be also manifested against Gram-positive bacteria. The *S. aureus* strain was selected for testing since these bacteria are associated with a significant number of cases of staphylococcal food-borne disease [51].

*S. aureus* was more resistant to CEO vapor than Gram-negative *S. typhimurium* (Figure 3). *S. aureus* was more resistant to MEO components, based on the MIC values at 37 °C [44]. MEO vapor inhibited *S. aureus* growth the most efficiently at 37 °C, less efficiently at 25 °C, and the least efficiently at 4 °C (Figure 3b). At 25 °C and 37 °C, the MEO vapor suppressed the growth of *S. aureus* more efficiently than it suppressed the growth of *S. typhimurium*, whereas at 4 °C, MEO acted dramatically stronger against *S. typhimurium*. The CEO vapor acted similarly against both bacteria, and only acted slower against *S. aureus*. No viable *S. aureus* cells were observed after 48 h at 37 °C and after 120 h at 25 °C, and at 4 °C, the growth inhibition was weak and resembled that of a control sample at 4 °C (Figure 3b). The vapors of both EOs acted the least efficiently against *S. aureus* at 4 °C, acted better at 25 °C, and acted best at 37 °C. CEO is more efficient than MEO at 25 °C and 37 °C against both bacteria.

There are not many publications describing the EO vapor antibacterial efficacy. However, there is evidence that the MBC of cinnamaldehyde vapor for *Salmonella* sp. is 0.25 μL/mL, and for *S. aureus*, the MBC is 0.5 μL/mL at 37 °C, confirming that *Salmonella* is more susceptible to the vapor of the main CEO component [49]. Since EO components directly interact with the cell membrane, the most likely explanation for the difference between the sensitivity to MEO vapor between *S. typhimurium* and *S. aureus* at 4 °C is determined by the differences in the cell envelope structure and composition of the bacteria and may also involve other cell-specific traits [17,52]. Menthol, the main constituent of MEO, is related to the sense of cold because it activates the cold-sensing receptors in humans [53]. However, no such receptors are described in the bacteria, despite the fact that menthol is an inducer of plasmid loss in bacteria [54]. This phenomenon, along with the antibacterial mechanism of menthol, requires a deeper investigation. Apart from the MEO effects on the viability of *S. typhimurium* cells, our results are consistent with the general principle that more EO evaporates at higher temperatures; thus, the killing of bacteria is faster at higher temperatures [55]. However, the actions of EOs are not always predictable, even based on the boiling temperatures of the components; the mechanisms of EOs are more complicated and still require elucidation [16]. CEO vapor is more effective than MEO in the long-term *S. typhimurium* and *S. aureus* exposure at temperatures of 25 °C and 37 °C.

### 3.5. Effects of CEO and MEO Vapors on S. typhimurium-Infected Chicken Skin

**Figure 4:**
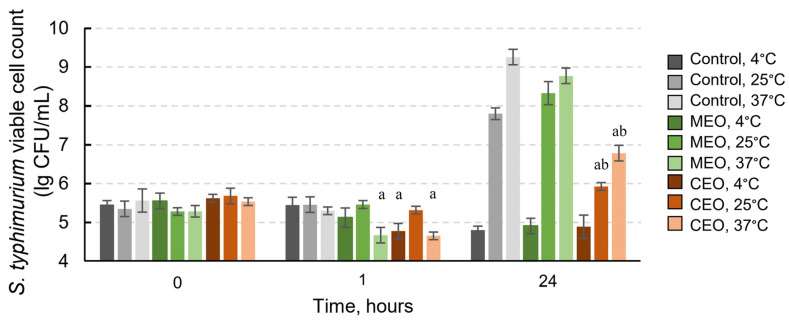
Viability of *S. typhimurium* cells on the surface of chicken skin after exposure to vapors of MEO and CEO at 4 °C, 25 °C, and 37 °C. Significant differences (*p* < 0.05) between the viable bacteria in the control and the EO-treated samples at the same timepoint and temperature are marked as a; significant differences between the samples of CEO and MEO are marked as b. All statistical analysis data are provided in Table S4.

We aimed to investigate the effects of MEO and CEO vapors against *S. typhimurium* located on the surface of chicken skin. Three temperatures, 4 °C, 25 °C, and 37 °C, were tested. After 1 h of incubation, the viability of *S. typhimurium* cells in the control samples was similar regardless of the temperature. Meanwhile, the viability of the cells significantly decreased from 5.5 logs (in control) to about 4.6 logs in the samples with EOs: the viability decreased at 4 °C with CEO (*p* = 0.025) and at 37 °C for MEO (*p* = 0.021) and CEO (*p* = 0.003) (Figure 4; Table S4). At 25 °C, the number of viable cells was similar in the EO-treated and control samples.

After 24 h of incubation, the live cell counts increased with the rise in temperature and reached the greatest values at 37 °C in the control samples and in the samples with EOs (Figure 4). Like in the in vitro testing of the EOs’ activity against *S. typhimurium*, CEO was more effective than MEO. The number of surviving cells significantly decreased under 24 h of treatment with CEO compared with the control and MEO samples at 25 °C (*p* = 0.0002 for both) and 37 °C (*p* = 0.0003 and *p* = 0.0006, respectively). An exposure to CEO reduced the count of *S. typhimurium* cells by 1.8 logs and 2.4 logs at 25 °C and 37 °C, respectively (Figure 4; Table S4). Thus, CEO performed better than MEO in inhibiting the growth of *S. typhimurium* on chicken skin, and it is more prominent at longer exposure times and higher temperatures of 25 °C and 37 °C.

According to the published data, *Salmonella* bacteria can grow on raw chicken skin at temperatures of 10–40 °C, whereas the growth at temperatures below 10 °C is not well understood [48,56]. Several EOs have been tested for their abilities to prevent the proliferation of *Salmonella* sp. bacteria on chicken meat and its products, namely bamboo, basil, lemongrass, thyme, orange, oregano, and white mustard [57,58,59,60,61,62]. Lemongrass EO, turmeric EO, and CEO have been used in the production of materials for food packaging to protect chicken meat [63,64,65]. Altogether, our work confirms that MEO and CEO are powerful natural oils that can effectively inhibit the growth of *S. typhimurium* via direct contact and via vapor, but the efficacy of this protection strongly depends on the EO concentration, contact method, time, temperature, and testing medium. The knowledge of CEO and MEO antibacterial properties in various conditions may aid in the development of novel strategies for the antibacterial protection of food products, especially chicken meat.

## 4. Conclusions

MEO consisted mostly of menthol (68.61%), while in CEO, (E)-cinnamaldehyde (83.32%) was dominant. Our findings show that in most conditions, CEO possesses stronger antibacterial properties against *S. typhimurium* than MEO. CEO exhibited a greater inhibition of *S. typhimurium* in both the agar diffusion and vapor diffusion assays in agar plates, whereas only at 25 °C, MEO vapor showed a stronger inhibition than CEO. *S. typhimurium* incubation in direct contact with EOs showed a similar antibacterial potential for both oils, since at 37 °C, the MICs were the same for both (20.83 µL/mL), but MEO remained a weaker alternative because of a two-fold higher MBC value (41.67 µL/mL) than that of CEO. However, MEO surpassed CEO in *S. typhimurium* killing via direct contact with diluted EOs at a longer exposure time and higher temperature (40 min; 37 °C). Also, MEO vapor showed a higher suppression of *S. typhimurium* at 4 °C than CEO in vitro, but no such effect was detected for Gram-positive *S. aureus* bacteria or in the experiments on the contaminated chicken skin samples. CEO was more effective than MEO at slowing down the proliferation of *S. typhimurium* on chicken skin at 25 °C and 37 °C, but for 4 °C, neither of the storage vapors of the tested oils had a role in controlling bacterial growth in a 24 h period.

## Figures and Tables

**Table 1:** Information on the origins of the EOs used in this study.

	Wild Mint EO (MEO)	Chinese Cinnamon Bark EO (CEO)
Botanical name	*Mentha arvensis*	*Cinnamomum cassia*
Batch number	LOT: MA02-06-18	P244801
Country of origin	Nepal	Australia
Best before	June 2023	February 2026

**Table 2:** Main composition (%, including constituents with quantity above 1.0%) of MEO and CEO. A full list of components is available in Supplementary Materials (Table S1).

Compound Name	RI_Lit_/RI_Exp_	MEO, %	CEO, %
α-pinene	939/939	1.07	0.10
β-pinene	979/978	1.06	0.04
Limonene	1029/1030	2.73	0.07
**Menthone**	1152/1152	7.9	
**Isomenthone**	1162/1162	6.42	
**Menthol**	1171/1172	68.61	
Geraniol	1252/1258	2.01	
**(E)-cinnamaldehyde**	1270/1271		83.32
Menthyl acetate	1295/1295	2.1	
(E)-cinnamyl acetate	1446/1445		4.69
**(E)-methoxy-cinnamaldehyde**	1528/1529		7.62
Total		91.90	95.86

RI_Lit_: Kovat’s indices for non-polar column, DB-5, taken from [35]; RI_Exp_: Kovat’s indices determined experimentally on the non-polar column, Rxi-5MS (Restek, USA); bolded compounds are those whose amounts were more than 5%.

## Data Availability

Data will be made available upon request.

## Supplementary Materials

The following supporting information can be downloaded at https://www.mdpi.com/article/10.3390/foods12213938/s1, Table S1: Total composition of MEO and CEO; Table S2: *S. typhimurium* bacteria counts (lg CFU/mL) after incubation with MEO and CEO at different temperatures. Each EO was premixed with 75% ethanol at a 1:1 ratio (*v*/*v*). MIC of each EO was diluted 10-fold, 100-fold, and 1000-fold with LB broth. Table S3: Viability of *S. typhimurium* and *S. aureus* affected by MEO and CEO vapors at different temperatures; Table S4: Viability of *S. typhimurium* on chicken skin after exposure to MEO and CEO vapors at different temperatures.
