# Supplementary material for: Essential Oils of Mentha arvensis and Cinnamomum cassia Exhibit Distinct Antibacterial Activity at Different Temperatures In Vitro and on Chicken Skin
Source: Foods. 2023 Oct 27;12(21):3938. doi: 10.3390/foods12213938 (PMC10647671; doi:10.3390/foods12213938)
Supplement: Supplementary file 1 [file foods-12-03938-s001.zip › Table S1.pdf]

Supplementary material

Table S1. Total composition of MEO and CEO.

No	Compound name	$RI_{Lit.}/RI_{Exp.}$	MEO, %	CEO, %
1	Tricyclene	927/929	tr.	
2	*α*-Thujene	930/930	0.02	
3	*α*-Pinene	939/939	1.07	0.10
4	Camphene	954/955	0.02	0.07
5	Thuja-2,4(10)-diene	960/659	tr.	
6	Benzaldehyde	960/960		0.10
7	Sabinene	975/974	0.32	
8	*β*-Pinene	979/978	1.06	0.04
9	3-Octanone	983/983	0.01	
10	6-methyl-5-Hepten-2-one	985/985	0.01	
11	Myrcene	990/991	0.56	
12	Ethyl hexanol	998/996	0.48	
13	*para-Mentha*-1(7),8-diene	1002/1001	0.07	
14	*α*-Terpinene	1018/1017	0.01	
15	*para*-Cymene	1024/1024	0.04	0.08
16	Limonene	1029/1030	2.73	0.07
17	1,8-Cineole	1031/1031	0.18	
18	(*Z*)-*β*-Ocimene	1039/1040	0.02	
19	(*E*)-*β*-Ocimene	1050/1050	0.02	
20	Bergamal	1050/1051	tr.	
21	*γ*-Terpinene	1059/1059	0.02	
22	(*Z*)-Sabinene hydrate	1070/1071	0.01	
23	(*Z*)-Linalool oxide	1071/1072	0.05	
24	Terpinolene	1088/1088	0.03	
25	*para*-Cymenene	1091/1092	0.01	
26	Linalool	1096/1096	0.22	
27	(*Z*)-Rose oxide	1108/1109	tr.	
28	*β*-Thujone	1114/1118	0.01	
29	(*Z*)-*para*-Menth-2-en-1-ol	1124/1124	0.02	
30	(*E*)-Rose oxide	1125/1125	0.01	
31	(3*Z*)-Hexenyl isobutanoate	1146/1146	0.02	
32	Isopulegone	1149/1150	0.93	
33	**Menthone**	1152/1152	**7.90**	
34	**Isomenthone**	1162/1162	**6.42**	
35	Borneol	1169/1169		0.23
36	**Menthol**	1171/1172	**68.61**	
37	*α*-Terpineol	1188/1188	0.41	
38	Decanal	1201/1200	0.10	
39	Octyl acetate	1214/1214	0.11	
40	4-methyl-dec-3-en-5-ol	/1216	0.08	
41	(*E*)-Carveol	1216/1219	0.03	
42	Citronellol	1225/1225	0.48	
43	(3*Z*)-Hexenyl 2-methyl butanoate	1232/1232	0.51	
44	Pulegone	1237/1236	0.37	
45	Carvone	1243/1243	0.02	

46	Geraniol	1252/1258	2.01	
47	Geranial	12681268	0.23	
48	**(*E*)-Cinnamaldehyde**	1270/1271		**83.32**
49	Neomenthyl acetate	1271/1272	0.15	
50	Lavandulyl acetate	1271/1273	0.04	
51	Menthyl acetate	1295/1295	2.10	
52	Carvacrol	1299/1300	0.02	
53	Geranyl formate	1300/1301	0.01	
54	Isomenthyl acetate	/1338	0.11	
55	Dihydro carveol acetate	1344/1343	0.04	
56	Citronellyl acetate	1350/1350	0.18	
57	Eugenol	1359/1359	0.04	
58	Cyclosativene	1371/1370		0.05
59	Unknown	/1371		0.19
60	*α*-Copaene	1375/1375	0.01	0.75
61	Geranyl acetate	1379/1379	0.18	
62	(3*Z*)-Hexenyl-(3*Z*)-hexanoate	1380/1380	0.01	
63	*β*-Bourbonene	1388/1388	0.17	0.02
64	*β*-Elemene	1390/1390	0.09	0.02
65	Decyl acetate	1404/1404	0.11	
66	*α*-Cedrene	1411/1409		0.01
67	(*Z*)-*α*-Bergamotene	1416/1416		0.02
68	(*E*)-Caryophyllene	1419/1419	0.40	0.18
69	*β*-Copaene	1432/1433	0.03	
70	(*E*)-*α*-Bergamotene	1434/1434		0.07
71	Aromadendrene	1440/1440		0.03
72	(*E*)-Cinnamyl acetate	1446/1445		4.69
73	*α*-Humulene	1454/1454	0.05	0.02
74	*β*-Santalene	1459/1459		0.03
75	Alloaromadendrene	1460/1460		0.05
76	*γ*-Muurolene	1477/1478	0.02	0.17
77	*α*-Curcumene	1480/1480		0.11
78	Germacrene D	1481/1483	0.18	
79	*β*-Selinene	1490/1490		0.03
80	10,11-epoxy-Calamenene	1492/1491		0.02
81	Viridiflorene	1496/1467		0.09
82	Bicyclogermacrene	1500/1500	0.02	
83	*α*-Muurolene	1500/1500	0.02	0.14
84	*β*-Bisabolene	1505/1503		0.19
85	*γ*-Cadinene	1513/1512	0.05	0.11
86	*δ*-Cadinene	1523/1523	0.07	0.28
87	(*E*)-*γ*-Bisabolene	1528/1528		0.16
88	**(*E*)-*methoxy*-Cinnamaldehyde**	1528/1529		**7.62**
89	*α*-Cadinene	1538/1538	0.01	
90	*α*-Elemol	1549/1549	0.07	
91	(*E*)-Nerolidol	1563/1563	0.03	0.10
92	Spathulenol	1578/1578	0.04	0.10
93	Caryophyllene oxide	1583/1583	0.09	0.08
94	Tetradecanal	1612/1612		0.04
95	*1,10-di-epi*-Cubenol	1619/1620		0.03

96	γ-Eudesmol	1632/1632	0.01	
97	Caryophylla-4(12),8(13)-dien-5α-ol	1636/1636		0.02
98	T-Muurolol	1642/1642	0.02	0.04
99	α-Muurolol	1646/1645		0.01
100	β-Eudesmol	1645/1646	0.01	
101	α-Cadinol	1650/1650	0.04	0.02
102	Agerochromene	/1662	0.01	
103	α-Bisabolol	1685/1686		0.04
104	Rimuene	1896/1895		0.06
105	Luxuriadiene	2006/2010		0.06
	**Total**		**99.66**	**99.10**

RI_{Lit}: Kovat's indices for non-polar column DB-5 taken from the literature [35]; RI_{Exp}: Kovat's indices determined experimentally on the non-polar column Rxi-5MS (Restek. USA); bolded compounds are those whose amounts were more than 5 %; tr. – trace amount, less than 0.01
