# Supplementary material for: Essential Oils of Mentha arvensis and Cinnamomum cassia Exhibit Distinct Antibacterial Activity at Different Temperatures In Vitro and on Chicken Skin
Source: Foods. 2023 Oct 27;12(21):3938. doi: 10.3390/foods12213938 (PMC10647671; doi:10.3390/foods12213938)
Supplement: Supplementary file 1 [file foods-12-03938-s001.zip › Table S2.pdf]

Table S2. *S. typhimurium* bacteria counts (lg CFU/mL) after incubation with MEO and CEO at different temperatures. Each EO was premixed with 75% ethanol at a 1:1 ratio (v/v). MIC of each EO was diluted 10-fold, 100-fold, and 1000-fold with LB broth.

MEO

	**4°C**			**25°C**			**37°C**		
**min** / **fold**	10	20	40	10	20	40	10	20	40
1	0.00 ± 0.00	0.00 ± 0.00	0.00 ± 0.00	0.00 ± 0.00	0.00 ± 0.00	0.00 ± 0.00	0.00 ± 0.00	0.00 ± 0.00	0.00 ± 0.00
10	6.90 ± 0.02	0.00 ± 0.00	0.00 ± 0.00	6.36 ± 0.02	0.00 ± 0.00	0.00 ± 0.00	6.35 ± 0.05	0.00 ± 0.00	0.00 ± 0.00
100	7.61 ± 0.02	7.55 ± 0.01	7.33 ± 0.04	7.61 ± 0.01	7.34 ± 0.03	6.65 ± 0.02	7.61 ± 0.02	6.79 ± 0.01	0.00 ± 0.00
1000	7.61 ± 0.01	7.52 ± 0.04	6.94 ± 0.01	7.61 ± 0.02	7.65 ± 0.03	7.49 ± 0.04	7.61 ± 0.02	7.40 ± 0.01	3.09 ± 0.07

CEO

	**4°C**			**25°C**			**37°C**		
**min** / **fold**	10	20	40	10	20	40	10	20	40
1	0.00 ± 0.00	0.00 ± 0.00	0.00 ± 0.00	0.00 ± 0.00	0.00 ± 0.00	0.00 ± 0.00	0.00 ± 0.00	0.00 ± 0.00	0.00 ± 0.00
10	6.90 ± 0.02	0.00 ± 0.00	0.00 ± 0.00	6.91 ± 0.01	0.00 ± 0.00	0.00 ± 0.00	6.91 ± 0.01	0.00 ± 0.00	0.00 ± 0.00
100	7.67 ± 0.01	7.50 ± 0.03	6.90 ± 0.02	7.66 ± 0.02	5.26 ± 0.20	3.78 ± 0.14	7.67 ± 0.02	4.67 ± 0.15	2.93 ± 0.24
1000	7.60 ± 0.02	7.71 ± 0.03	7.79 ± 0.02	7.53 ± 0.06	7.53 ± 0.06	7.63 ± 0.05	7.62 ± 0.01	7.46 ± 0.04	7.85 ± 0.02
